# An implementation evaluation of a policy aiming to improve financial access to maternal health care in Djibo district, Burkina Faso

**DOI:** 10.1186/1471-2393-12-143

**Published:** 2012-12-08

**Authors:** Loubna Belaid, Valéry Ridde

**Affiliations:** 1 Research Centre of the University of Montreal Hospital Centre, CRCHUM, Montreal, Quebec, Canada; 2 Department of Preventive and Social Medicine, Faculty of Medicine, University of Montreal, 3875 Saint-Urbain St, Montreal, Quebec, Canada

**Keywords:** Implementation, Evaluation, Health policy, Micro interaction, Burkina Faso

## Abstract

**Background:**

To bring down its high maternal mortality ratio, Burkina Faso adopted a national health policy in 2007 that designed to boost the assisted delivery rate and improving quality of emergency obstetrical and neonatal care. The cost of transportation from health centres to district hospitals is paid by the policy. The worst-off are exempted from all fees.

**Methods:**

The objectives of this paper are to analyze perceptions of this policy by health workers, assess how this health policy was implemented at the district level, identify difficulties faced during implementation, and highlight interactional factors that have an influence on the implementation process. A multiple site case study was conducted at 6 health centres in the district of Djibo in Burkina Faso. The following sources of data were used: 1) district documents (n = 23); 2) key interviews with district health managers (n = 10), health workers (n = 16), traditional birth attendants (n = 7), and community management committees (n = 11); 3) non-participant observations in health centres; 4) focus groups in communities (n = 62); 5) a feedback session on the findings with 20 health staff members.

**Results:**

All the activities were implemented as planned except for completely subsidizing the worst-off, and some activities such as surveys for patients and the quality assurance service team aiming to improve quality of care. District health managers and health workers perceived difficulties in implementing this policy because of the lack of clarity on some topics in the guidelines. Entering the data into an electronic database and the long delay in reimbursing transportation costs were the principal challenges perceived by implementers. Interactional factors such as relations between providers and patients and between health workers and communities were raised. These factors have an influence on the implementation process. Strained relations between the groups involved may reduce the effectiveness of the policy.

**Conclusions:**

Implementation analysis in the context of improving financial access to health care in African countries is still scarce, especially at the micro level. The strained relations of the providers with patients and the communities may have an influence on the implementation process and on the effects of this health policy. Therefore, power relations between actors of the health system and the community should be taken into consideration. More studies are needed to better understand the influence of power relations on the implementation process in low-income countries.

## Background

Improving access to skilled attendance childbirth remains a major public-health challenge in Africa [1,2]. A set of factors has been identified as barriers to healthcare access: sociocultural factors, perceived benefits and needs, physical and financial accessibility [3,4]. Financial limitations were noticed as an important barrier to access to maternal health care [3].In the context of achieving the Millennium Development Goals, African countries have engaged in political actions to minimize this factor by eliminating user fees.

User fees were seen as an effective solution for funding improved primary health care [4,5]. More than two decades after the implementation of user fees, the objective of improving access to primary health care has still not been achieved; in fact, they are now considered a major barrier to accessing health services. In this context, many countries have decided to eliminate user fees. The literature documenting these policies has focused mainly on effects [6]. However, few articles have been found focusing on the modalities of implementation [6]. Six issues surrounding the implementation process of removing user fees were raised: the importance of having sufficient funding, maintaining adequate drug stocks, and involving front-line workers in the decision-making process [6]. Providing incentive measures was seen as essential to improve staff morale [7,8]. Improving communication strategies to inform the general population was identified as one of the key points in the implementation process [9,10]. Finally, improving the provider-patient relationship was considered a key influence on the implementation process and its effectiveness [9].

In a literature review analyzing health policies in low- and middle-income countries, Erasmus and Gilson (2008) indicated that this area of research *“remains in its infancy”*[11]. Yet analysis of the implementation process at the micro level is essential to highlight factors that can counteract or facilitate the implementation of the intervention [12-19]. However, this issue in the context of improving financial access to maternal health care has received little attention in the literature to date [11]. This paper presents an implementation evaluation of a national maternal health policy that aims to subsidize the cost of deliveries and emergency obstetric care in a district of Burkina Faso. Two evaluations were carried out on the implementation of this national health policy. There are previous reports of short-term analyses that identified weaknesses in this implementation, but they do not document in detail interactions between participants at the micro level that may have an influence on the effects of this policy [20,21]. In order to explore the issues in greater depth, the objective of this paper is to analyze the perceptions of this health policy by health workers and the implementation of this policy at the district level, and also highlight interactional factors that have an influence on the implementation process.

### Intervention background

Burkina Faso is a West African country with 16 million inhabitants (World Bank, 2010). The maternal mortality ratio is estimated at 560 maternal deaths per 100.000 live births (WHO, 2008). Health system is composed of two levels. The first level is the health centres (CSPS) which cover several villages. A team of health workers (two nurses and a skilled birth attendant) manage this level of care. They provide basic outpatient, inpatient curative and preventive care, including vaccinations and antenatal care. They also provide basic emergency obstetric care (EMOC), which includes antibiotics, oxytocics and convulsants, manual removal of the placenta, assisted vaginal delivery and newborn care [22-24].

Health workers receive bonuses based on their medical activities. They are entitled to 20% of medical consultations and actions per patient [25]. According to a study published in 2009, the net salary of nurses with less than 5 years experience working in the public sector is estimated at 110.409 CFA (168 Euro) (median amount) [25].

Community management committees (COGES), which are elected by the population, are responsible for the financial management of these health centres. They are responsible for the revenues generated from user fees (for curative care, hospitalization) and from subsidies for delivery and emergency obstetric care. The second level is the district hospitals (CMA), which are located in towns. They serve the population of the entire district and all emergency cases from CSPS. There is a surgery department in the district hospital and a blood transfusion service. This level provides comprehensive emergency obstetric care, which means all the basic emergency obstetric care plus caesarean sections and care to sick and low birth weight newborn [22-24]. The third level of the health system is the regional hospitals.

Traditional birth attendants (TBAs) performed deliveries until 2007 [26]. Today, their role is to help women to reach a health centre. However, they still perform deliveries in some villages that are far from a health centre. In some health centres, they are invited to participate informally in activities (e.g. increasing awareness, cleaning the delivery room). In some health centres, they receive a bonus fee. In the context of maternal health services, TBAs and community health workers play the same informal role (helping women to reach a health centre to give birth, increasing awareness). However, TBAs are more inclined to perform deliveries.

### Intervention

**Figure 1:**
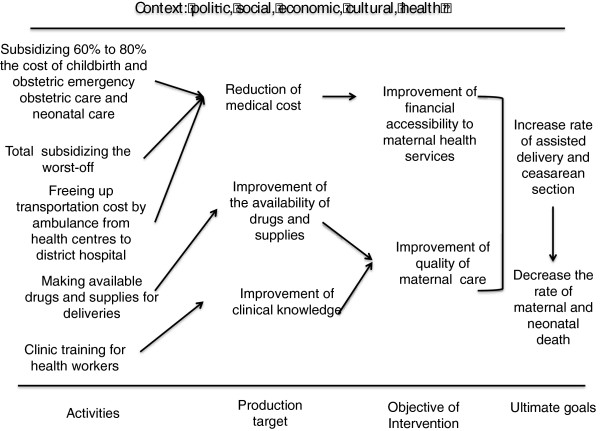
Logic model of the national subsidy of obstetric care

Figure 1 summarizes the process by which this policy aims to increase the rate of assisted delivery by acting on economic accessibility. It describes activities, available resources, intended objectives and goals that this policy aims to reach. The subsidy policy targets pregnant women. This health policy was approved by the council of ministers in April 2006. It consists of subsidizing 60% to 80% of the cost associated with assisted deliveries and emergency obstetric care depending on the level of care. Women pay the remainder [22]. For example, for an uncomplicated delivery at a health centre, women have to pay 900 CFA (1.4 Euro).

For the worst-off, the policy provides a full exemption. However, the management mechanism to deal with this population group has yet to be defined.

For caesareans and emergency obstetric care, the policy supports the cost of transportation from the health centre to the district hospital. Women are fully exempted from this cost. At the district level, financial resources allocated by the central level for the cost of transportation are managed independently from subsidies for deliveries and emergency obstetric care.

Financial resources are directly allocated to health districts. District health managers carry out resource allocation for health centres by giving them a bank check from their financial institution. The amount given is based on the volume of services provided to the target population. These resources reimburse the cost of drugs and supplies used for deliveries. Health centres are reimbursed up to 3600 CFA (5.5 Euro) for each uncomplicated delivery performed, and up to 14.400 CFA (22 Euro) for each dystocic labour. The main source of funding is the state budget.

## Method

Multiple case studies were conducted between November 2010 and February 2011 in six health centres in the district of Djibo, located in the province of Soum in Burkina Faso. This district was chosen because it is part of a larger research program on the effect of the elimination of user fees at point of service on the use of health services [27]. In this research program, the district of Djibo was used as a control district, since only the national subsidy of obstetric care was applied (no intervention of NGOs freeing up health care). Therefore, the data quality of the HIS (Health Information System) of the MoH (Ministry of Health) was verified and due to the context of the program (national policy implementation without NGO support), the district of Djibo was chosen to conduct the study. Health centres were selected based on a list of criteria’s including geographic accessibility and localization, social and cultural environment, type of health centre (isolated clinic, maternity), health attendants (presence or absence of skilled childbirth attendant), and rates of curative consultations, prenatal and assisted deliveries [28].

The data sources described in Table 1 (documents, interviews, focus groups, observations and feedback session on the results) were used to boost the validity of this study through triangulation [29].

**Table 1:** Methods and sources of data

**Methods**	**Sources of Data**	**Sample size**	**Variables examined**
In Depth interviews	Perceptions of the policy (content, implementation, effects)		History of the implementation; challenges and difficulties information communication, process of funding, relation between stakeholders, quality of care
	District Health Managers	10	
	Health workers	16	
	COGES	11	
	TBAs	7	
Focus Groups	Patients, Communities (men; CHW, TBA)	62 (8 to 10 persons for each focus group)	Partograph, audits of maternal deaths, costs associated with childbirth (formal and informal payments) barriers to access to health care, quality of care
Documents	Maternal deaths audits, guidelines, register of birth, sheet management, ethnography of the locality	21 audits of maternal deaths 1 guideline registers of birth from 6 health center from 2007 to 2010	Causes of maternal deaths, delays, distance between health centres and district hospital, timing of the ambulance, components of the health policy, process of funding, tasks and responsibilities of each actor, number of births in health centres
		2 ethnography of the locality	
Observations	Curative and prenatal consultations, assisted deliveries, vaccinations, distribution of nets	Observations were carried out in 6 health centres (from 7 to 10 days were spent in each health centre)	Interaction between health workers and communities (patients, communities, CHWs, TBAs)

*COGES*: Community management committee; *TBA*: Traditional birth attendants; *CHW*: Community health workers.

### Individual interviews and focus groups

Four categories of participants (district health managers, health workers, TBAs and members of (COGES) that are considered to be key personnel in the implementation process were interviewed [12]. The aim of these interviews was to understand their perceptions of the national policy and the history of challenges associated with the implementation process. All interviews were recorded. The first author and a local assistant conducted the interviews and focus groups. Topics covered in the interviews are listed in Table 1. Notes were taken for each interview. For each health centre, health personnel, the COGES and the TBAs from different villages covered by the health centres were interviewed. In total, 16 interviews were carried out with health workers, 10 with district health managers, 11 with COGES members and 7 with TBAs (Table 1).

The interviews with TBAs and communities were conducted in the local language (Fulfulde) and then translated into French. Two local translators translated independently in order to ensure that the unstructured guide was accurately translated.

Focus groups were organized to document the perception of this new healthcare policy, amounts paid for delivery costs, coping strategies to pay for medical costs and transportation, availability of information about this policy, and perceptions of quality of care at the health centre. In total, 62 focus groups were conducted with communities in villages. All the principal villages covered by the six health centres were targeted for focus groups. This represents between 5 and 15 villages per health centre. Women and men were met separately so that they would feel more comfortable talking about maternal health. Focus groups were formed to include individuals who had used maternal health services during the implementation of the policy and individuals who had lived in the village for several years in order to obtain a wide range of perspectives on quality of care provided by their health centre. Community health workers were asked to gather a group of 8 to 10 women and 8 to 10 men in the villages. Men and women were gathered in the public square of the village. The selection process involved gathering participants who share common characteristics (people who live in villages, agriculture and husbandry as main economic activities) in relation to the topic (the national subsidy of obstetric care). The objective of the selection was to mix opinions in order to highlight points of view on this national subsidy of obstetric care and relations with the health centre.

Community health workers and traditional birth attendants were not systematically present during the focus groups. However, data do not show any difference when they were present or absent. We made sure that all participants could express their ideas, views on the national subsidy for obstetric care and relationships in the health centre. The transcript of focus groups was written in French.

### Analysis of documents

Twenty-three documents (maternal death audits, guidelines, management sheets, register of birth, ethnography) were screened and analyzed to better understand the history of this maternal health policy and the context in which it was implemented [28].

### Observations

Non-participant observations were conducted in each health centre (N = 6) to examine the interactional aspects between health workers and communities and assess how these interactions influence the implementation process. Notes of these observations were taken. Lengthy immersion in the field (4 months) by the first author was undertaken to understand the social context of the study. The first author conducted the observations in each health centre, spending 7 to 10 days at each health centre. The second author, who has worked in this area of research for 12 years, has in-depth knowledge of this maternal health policy.

### Feedback session on the findings

Preliminary results were presented and discussed with the members of district management and 20 health workers in the district in February 2011 in the presence of the two authors. Work groups were organized, and the participants were asked about their perceptions of the results, whether they agreed or disagreed with the findings of the study, and their ideas on how to improve the efficiency of this maternal health policy. The whole session was recorded and transcribed. Several authors have reported that sharing the results of a study with stakeholders and allowing them to participate in the evaluation process is very beneficial for understanding the phenomenon under study and finding solutions to improve the effectiveness of the intervention [13].

### Data analysis

All interviews and notes were transcribed and codified with the qualitative software QDA Miner. A thematic approach was used [29]. A list of themes (subsidy, transportation, quality of care, monitoring, effects) was drawn up, based on the logic model (Figure 1), the objectives of the study and observations from the field. Each interview was codified according to these themes in order to observe the similarities, contrasts and outliers of what was said for each theme [29].

### Ethical considerations

The health research ethics committees of the Ministry of Health of Burkina Faso (no. 2010–072) and the University of Montreal (CRCHUM) approved the study (10.178). Authorization (2010 06 04 MS-RSHL-DRS) was given by the direction of the health region in the Sahel to conduct the study. The anonymity of participants was maintained throughout the study. Informed consent was obtained from the participants.

## Results

Results will be presented as follows: the study site, perceptions of the health policy (content and implementation), the implementation process, and the interactional factors that were found to have an influence on this maternal health policy.

### Study site

This district with 390.000 residents is located in the province of Soum in Burkina Faso [30].

Composed of rural departments, it is one of the poorest regions in Burkina Faso. The 193 villages in this region are unevenly distributed. There are 31 health centres (one per 12.500 people vs. a national average of one per 10.000) and one district hospital. Twenty-seven health centres do not meet infrastructure standards and 10% of health centres do not meet human resource standards [30]. The average distance between villages and health centres is estimated at 11.4 km [30]. In 2009, the rate of assisted delivery was estimated at 53.2% [30].

### Perceptions of the health policy

Health workers welcomed this national health policy. First, they see it as a good strategy for improving access to health care, both to increase the rate of antenatal care and as an opportunity to further extend the intended results into other areas, such as a higher vaccination rate. However, health staff stated that this national health policy should cover the whole process of pregnancy, not only the delivery, in order to prevent complications. They indicated that they are aware that this national health policy is not enough to effectively decrease maternal mortality. From their perspective, other measures should be added. Comment from a district health manager: “*This health policy alone cannot meet the challenges of access to health care. We need to reduce distances between villages and health centres, and change people’s behaviour.”*

The implementation process was perceived to be difficult because the guidelines were not clear in terms of targets, extension (period of time) and drugs covered by the policy. Therefore, at the beginning of the implementation there was some variation in the understanding of the national policy, as noted by a nurse: *“Everybody is doing it his own way.”* (health centre 5).

### Implementation

The implementation analysis section was described according to the components of the health policy (Figure 1). For each component, the congruency of what was planned, what was implemented as per official records, and what was actually done as reported by participants was examined.

#### The reimbursement system

According to the policy guidelines, health centres can be reimbursed for two types of assisted deliveries: normal deliveries (no complications) and complicated deliveries (dystocic labour). For normal labour, health centres were reimbursed 3600 CFA (5.5 Euro) for each delivery performed. For complicated deliveries, health centres were reimbursed 14.400 CFA (22 Euro). According to the district health manager, the rate of dystocic labour in health centres has doubled since the beginning of the implementation of this health policy. To reduce these costs, the health district manager ordered staff to stop writing dystocic labour on the reimbursement sheet. Therefore, health centres are reimbursed only for uncomplicated deliveries. *“They re-defined dystocia for us. It is not the dystocia that we learnt at medical school. Money is involved in that”* (nurse, health centre 4)*. “We are told not to do any more dystocia labour. However, in health centres we deal with dystocia labour”* (nurse, health centre 2).

Two years after the launch of the policy, the reimbursement system was changed. Since 2009, health centres are no longer reimbursed at a fixed rate, but on the basis of actual expenses. Reimbursement sheets for the policy were consulted in the 6 health centres that were studied. The cost of normal deliveries ranged from 1200 to 1500 CFA (2 to 2.3 Euro) in health centres. Health district managers perceived the national level estimate of the cost of a normal delivery as being too high. Changes in the modalities of reimbursement in health centres were key turning points in the implementation of this health policy. According to the district managers, health workers asked for reimbursement of dystocic labour in order to have higher refund payments and a more generous budget: *“We found that it was for a greater balance.”*

The estimated cost per patient for a caesarean section, as planned in the policy, was 11.000 CFA (17 Euro). In 2008, health district managers decided to pay 5000 CFA (7.6 Euro) of this cost by drawing on the budget line for drugs provided by the state. In 2009, the remaining 6000 CFA (9 Euro) was paid by each COGES each month. Therefore, parturients do not pay for caesarean sections (which cost 11.000 CFA). This initiative came into effect in July 2010 and the impact remains to be studied.

#### The grant for the worst-off

The policy planned to fully exempt the poorest patients from all costs associated with assisted deliveries and emergency obstetric care [22]. A budget from the national subsidy obstetric care policy of 50000 million CFA (76.2 million Euro) was planned to fund the poorest patients. In the district of Djibo the subsidy for the worst-off was not implemented. *“The application is very hard, starting with the definition: what is a worst-off?” “Which criteria should we use to say that this person is a worst-off and this one is not?”* (district health *manager*)*.*

#### Transportation

The cost of transportation between a health centre and the district hospital is covered by the policy. The transportation funding was supposed to be managed separately from the budget for deliveries and emergency obstetric care. The main problem perceived by the district is the long delay in reimbursement for transportation from health centres to the district hospital. The outstanding amount, supported by the district hospital, is estimated at 6 million CFA (91.500 Euro). According to the health district managers, this is leading to financial difficulties. The delay is due to misunderstanding the process of reimbursement by the district health manager. The district health manager does not keep receipts from the purchase of gasoline; however, this ticket is the basis on which the reimbursement is made.

The district of Djibo covers an area of 12.700 km^2^[30]. Only one ambulance is functional for the whole district, which leads to significant delays in referrals, as outlined by one health worker: *“You can call for the ambulance and find that it is elsewhere, so you must wait for the ambulance to come back out. It is a real problem”* (health centre 3). The ambulance is used frequently and it is a multi-purpose pick-up vehicle, which is also used for other emergency transportation.

#### Quality of care

The implementation guidelines for this policy recommend improving the quality of care [22]. The quality of care component in the guidelines was composed of various activities, such as continuity in conducting maternal death audits, and the implementation of a quality assurance service (QAS) team in the district hospital in charge of safety and enforcement of hygiene standards. Health workers are supposed to conduct surveys of patients to assess quality of care in health centres. For each delivery, health staff is supposed to use a partograph to assess the process of delivery. Health workers are informed and trained to write maternal death audits, these audits are carried out in the district of Djibo, and the records of these were available for study. Sessions to discuss maternal death audits were organized at the district hospital. However, the surveys of the beneficiary population and the QAS team were not implemented. *“In our health centre we did not implement these activities. The survey that we should do, we learnt that from you [the researcher]. They did not give us any guidelines or document that talks about that”* (nurse at health centre 1).

In health centres where there are no midwives or skilled birth attendants, the partograph is not used. These centres represent 13.3% of health centres in the district of Djibo [30]. Problems using the partograph reported by health workers are related to both social context and working conditions in health centres. The patients often come to the health centre when they are fully dilated or in the expulsion phase of labour, while the start of writing a partograph should begin at four centimetres dilation. As noted by a skilled birth attendant (health centre 1)*: “Women usually come to the health centre when they are about to give birth. It is then difficult to open a partograph to monitor.”* The lack of infrastructures (no electricity) also makes using partographs difficult. A skilled birth attendant said that she stopped opening partographs because she refused to work with a flashlight (health facility 1); 24% of health centres do not have electricity [30].

#### Supplies and equipment

One of the key components of the policy guidelines is to make drugs and supplies available for performing deliveries. Although there have not been any significant disruptions in drugs and supplies, items are not sufficiently available in the delivery room, as indicated by a midwife: *“In health centres there were no gloves for revision* [*of the uterus*]*, while you are often asked to make revisions, you are forced to tinker”* (Health centre 6). In addition, equipment such as birth boxes and delivery tables is limited and heavily used, resulting in problems with providing good quality care. As a health district manager has indicated: *“the material is not enough. This material wears out quickly.”*

#### Incentive measures

The policy as planned did not provide a specific incentive system for health workers in addition to the usual (20% bonus per procedure). The system of bonuses was added later in the district with the approval of the central level: *“Health workers thought that this policy came as a huge work load. They asked for a financial motivation for this policy. But it is written nowhere. We asked the central level, and we were told that is only on the medical act that you can take 20% bonus”* (district manager)*.* In the district of Djibo, this system is applied differently from one health centre to another. Some health centres have applied this 20% bonus only to medical procedures. Others have applied it to the contribution of the patients, and others have not applied it at all. According to health workers, this policy should have been planned to offer a formal financial incentive measure to encourage them in their work in addition to the 20% bonus.

#### Communication

In the district of Djibo, the process of informing the population of this health policy was vertical. The information came first to the district health manager and went through health workers, the local political community and finally to the communities. The main communication channels that were used were antenatal care consultations, routine vaccinations in villages, and the radio. However, many women were still not informed of this health policy: *“We did not know that the price of deliveries has changed. Nobody came to our village to tell us that”* (women’s focus group, health centre 1).

#### Monitoring system

Health workers have to fill in two documents related to this policy: individual patient records and the birth registry. At the beginning of the implementation, health workers had problems completing the individual patient record. “*Filling forms was a real problem because we did not understand some elements of the form to fill properly”* (nurse at health centre 3).

Document storage and entering the data in the electronic database mandated by the policy were the main administrative problems perceived by district health managers. The absence of an archiving system and the lack of space in the district caused more problems with storing all the reimbursement sheets for the 31 health centres that reach the district each month, as outlined by a health district manager: *“This policy has reiterated the problem of archiving in the district.”* There can be as many as 60 record sheets each month for a busy health centre.

The database software came one year after the implementation of the policy, creating a great delay in entering data in the database. In addition, the software is only available on one computer and cannot be duplicated on others. To overcome this problem, the health district manager hired a secretary from the district’s own budget to perform data entry. During the period of data collection for our study (November 2010), she was entering data from November 2009.

### Interactional factors

In this section, interactions between groups of participants at the community level were identified as having an influence on the implementation process, relation between patients and providers, health workers and TBAs, health workers and members of COGES. Poor relations between these groups may reduce the effectiveness of this policy.

#### An ambivalent relationship between health workers and patients

The implementation of this policy influences relations between health workers and patients. On the one hand, health workers perceived an improvement in relations, because they no longer have to negotiate medical costs with their patients. On the other hand, the misunderstanding surrounding the items covered by the policy leads to strained relations: *“The villager is what he is; he thinks that everything is free.”*(nurse, health centre 1) *“When patients come and there is nothing to pay, the atmosphere is more relaxed.”* (district manager).

However, some communities have not observed any changes in relations with health workers. In some villages the population is satisfied with the quality of care, whereas in others relations with their health workers are perceived as more negative. Informal payments, the rate of absenteeism, the unavailability of health workers, and the expression of authority over patients are the main issues highlighted by communities: *“When you go to pay for drugs at the pharmacy, they cheat all your money. I have had to pay drugs out there that rose from 35.000 (53.3 Euro) to 50.000 CFA”(76.2 Euro). “The skilled birth attendant did not assist me to deliver, she came when I had already delivered*”(health centre 1). Another women indicated*: “I decided not to go to the health centre to give birth because when we went for my co-wife, nobody was there to take care of her”* (health centre 2). Another woman reported: *“When you do something that did not fit, she [the skilled birth attendant] scolds you”* (health centre 1).

#### Traditional birth attendants and community health workers: forgotten by the policy

The national subsidy for obstetric care as planned did not involve traditional birth attendants, who had no assigned role under this national health policy. However, a decree from the Ministry of Health issued in December 2007 requires districts to redirect the role of TBAs [26]. Their role was shifted without any change in compensation. Nowadays, TBAs are asked to encourage women to attend health centres. Health workers and district managers have ambivalent perceptions of the role of TBAs and CHWs. On the one hand, they are seen as an obstacle to the policy because they continue to perform deliveries in villages against the aim of the policy. On the other hand, they are considered a potential ally to help with activities: *“Their presence is an advantage but also an inconvenience. They make our life difficult.”* (district health manager).

From the perspective of TBAs, they understand their new role but feel frustrated that they are not sufficiently involved in the activities of health centres*.* Moreover, as the new policy has transferred their former role of assisting during delivery to the health centre, this also means a loss of income for TBAs*. “My new role is good. Before we did not go to health centres and many women died after delivery, but since we started to go there is no maternal death but health workers do not invite me to their meetings. They did not give us anything (money).”* (TBA, health centre 2).

The perspective of CHWs is similar to that of TBAs. They feel useless and excluded from the activities of the health centre.

#### Relations between COGES and health workers: allies or enemies?

COGES play an important role in the implementation of the policy. Grant reimbursements are made from the district directly to their bank account. They are required to bear the costs until they receive the subsidy payments. Health workers perceive a lack of cooperation from these agents in the activities of the health centre, such as paying for gas to go to villages to raise awareness. According to health workers, COGES do not perform their role as an interface between the health centre and the community, for example in transmitting information. Problems in understanding the process of reimbursement lead to strained relations: *“They do not understand the modality of reimbursement, so when we ask them to pay for medication we have to negotiate with them.”* (nurse, health centre 3).

From the perspective of COGES, the long delay in reimbursement is the main difficulty perceived in the implementation of this national health policy, leading to the purchase of drugs on credit. Only one of the COGES members interviewed talked about negative relations between them and health workers, as stated by a COGES president: *“Most of the time the relationship doesn’t work. There is a lack of collaboration between health workers and us.”* (health centre 2).

## Discussion

Implementers have a positive perception of the content of this health policy. The lack of clarity in the guidelines has made the implementation of the policy difficult. The storage of documents, entering information in the database, problems with using the partograph, and the limited number of ambulances were the principal challenges faced by implementers. All the activities were implemented except for the grant for the worst-off and some activities related to quality of care. Interactional factors, such as relations between patients and providers and between health workers and communities, were raised.

### A fairly effective implementation, except for the lack of involvement of traditional birth attendants

In the context of improving financial access to health care, five issues surrounding the implementation process were raised: adequate funding, involving front-line workers in the decision-making process, providing incentive measures, improving the communications strategy, and quality of care [9,20,31]. In the district of Djibo, although reimbursement was estimated at a fixed rate, funding was adequate. Information on this health policy was provided vertically and in an executive manner. Incentive measures were partially implemented in health centres, with some but not all health centres establishing a bonus system. A lack of clarification on the way to calculate this bonus led to various payments. Although health workers perceived an improvement in provider-patient relations, the quality of care remains unchanged (Table 2).

**Table 2:** A fairly efficient implementation except for the lack of involvement of TBAs

**Issues**	**District of Djibo**	**Empirical data**
Adequate funding	Yes	Funding covers the expenses of assisted deliveries, no difficulties in the reimbursement system was perceived by district managers
Involving front line workers	No	Lack of involvement of health workers in the district, vertical process of transmission of information, execution implementation
Incentive measures	Yes, but partially	Variation in the application of the incentive measures, 3/6 of health centres have applied the incentive measure
Improvement of strategy of communication	No (remain unchanged)	Vertical process: from district to health centres to communities. Channels: prenatal care, vaccination sites, radio
Improvement of the relation between provider and patient	No (remain unchanged)	Informal payments, rate of absenteeism, health workers not available
Involving Traditional birth attendants and community health workers	No	Lack of involvement of TBAs, excluded from this national policy, loss of income for them
Making feed back session results with stakeholders	Yes	Work groups organized, discussion of the results, perceptions of the findings, strategies to improve the policy

The lack of involvement of traditional birth attendants and community health workers and their impact on the policy is an additional finding of this study. Both were negatively affected by the policy. Findings in this study are similar to research in Ghana, where 60% of TBAs felt that the policy of eliminating user fees has taken away their clients and caused them to lose part of their income [32]. Although they are no longer in charge of the health of the community, they do still play a role in the community and can influence the population, especially when they are present in villages that are far away from health centres [32-34]. Indeed, information on health care activities, such as vaccination campaigns, vitamin A supplement campaigns, antenatal care and new health policies is transmitted through them to the population.

In this sense, they have a direct influence on the process of implementation and the effects of the policy. Informing the population is one of the new tasks that were assigned to them. Because there is currently little evidence that their biomedical training as birth attendants is sufficient to allow them to reduce maternal mortality when performing their traditional role, their new role and their impact on maternal health are still being discussed [33,34]. Moreover, little information is available on the correlation between the role of TBAs and policies designed to improve assisted deliveries. More studies are needed to better understand the relationship between TBAs and the policy of eliminating user fees for health services.

### The benefits of evaluating the implementation process

Literature on the impact of fee removal policies on service utilization and out-of-pocket expenditures is growing [35-39]. However, implementation analysis is still scarce, specifically at the micro level, in the context of improving financial access to maternal care [6]. Yet it is essential for decision-makers who set up this type of policy, because it highlights barriers that counteract the implementation of such policies [12,13]. In the case of this study, implementation evaluation has identified the challenges that implementers face, such as problem with using the software for data entry, resulting in long delays in reimbursement.

In addition, implementation evaluation helps us to see the congruency between the planned and actual implemented intervention and understand the gap between the two [12]. In the case of this health policy, no great “*implementation gap*” was observed, except for the subsidy for the worst-off and for some activities related to quality of care [39]. Therefore, in the case of this policy, subsidies for the worst-off and improving quality of care should be targeted.

### Power relations within the implementation process

Screening interactional factors has led us to deeply examine micro interactions between participants in health-care settings. Analysis has shown the complexity of relationships between different groups of participants. One of the major findings is the daily practice of power by the people involved. As Walt (1994) stated, analyzing the implementation of health policies involves taking power relations into consideration [40]. Moreover, “*it is the sharp end of much health policy implementation,”* because it can modify the implementation process and may have an impact on the effects of a policy in reducing or enhancing the effectiveness of the policy [11]. In the case of this study, power relations were detected at 3 levels.

#### District health managers and health workers

According to Erasmus and Gilson (2008), the practice of power by central agents can be exercised by “*controlling frontline workers*” *or* by“*adapting intervention to local needs*” [11]. In this study, district health managers control front-line workers. The balance of power between these two groups shows a marked distinction of roles and tasks. Front-line workers perceived themselves as mere performers. By contrast, district health manager teams see themselves as “supervisors” or “controllers.” The terms “control,” “monitor” and “supervision” were used repeatedly to describe relations with health workers. These types of power relations emphasize the “*top-down model*” of implementation, where individuals in the upper level in the health system exercise their power based on authority given to them [11]. The implementation process for the health policy is done in a vertical process, where health workers are not involved in the decision-making process. In addition, health workers felt a lack of recognition from their superiors. This feeling leads to a crystallization of the relationship between these two groups of participants. Therefore, this type of power based on formal authority and hierarchy is known in organizations and institutions in low-income countries [41]. This type of power is rooted in the institutional culture of these settings [41,42]. In the context of implementing health policy, the question that we should ask is how to manage these structural relations of power. Although the agents in the upper sphere can use their power, *the street-level* workers who are at the bottom of the decision-making process also have discretionary power that they can exercise and thereby change the implementation process [43]. The discretionary power of health workers may be one of the reasons why the national subsidy of obstetric care failed to achieve its objectives [43].

#### COGES and health workers

In some health centres, COGES work and collaborate positively with health workers, but in most centres, the relationship is adversarial [44,45]. The central issue between the two groups of agents is the financial management of the health centre. The responsibilities of COGES have remained unchanged since the application of the Initiative of Bamako (IB). Financial management of this health policy has been added. Indeed, the refund policy is made directly from the district to the COGES bank account. Patients’ contribution is paid directly into their bank account and contributes to the purchase of drugs and supplies. Conflicts were palpable when it came to disbursing money for health centre expenses, as was the case even before the policy [15,44,45]. This finding is similar to two studies carried out in Mali and Senegal [15,44]. The difficulty of collaboration between COGES and health staff and the lack of transparency in the financial management of the health centre were highlighted in both studies. Power relations within the communities may weaken collaboration between the two groups of participants. In this research, misunderstanding of the reimbursement process outlined by the health policy enhances conflicts.

#### Providers and patients

The impact of provider-patient relations on the use of maternal health services in low-income countries has been documented [46-49]. It is not only an important factor in the choice to use maternal health services, but also a fundamental criterion for improving quality of care. In the context of policies mandating the elimination of user fees, relations between the provider and the patient remained unchanged [9,31,50], marked by the existence of informal payments demanded by health workers and misunderstanding of the policy [9,51]. This study supports this finding. Although health workers have seen an improvement in relations in the sense that they have no longer have to negotiate the payment of medical drugs, informal payments still exist. High rates of absenteeism for health workers in health centres and their unavailability and the expression of authority towards patients are “*routine practice*” in daily health care. According to Gilson and Erasmus, “*these routine practices”* could be considered forms of power by health workers over patients [11]. These power relations seem inherent in the health system, specifically in low-income countries. Therefore, in the context of implementing health policy, these power relations within the health system should be discussed.

### Limitations of the study

This study is one of the few research that analyze the implementation process for public health care policy at the micro level in the context of reducing the financial costs of maternal health care. The study was limited to one district due to budget and time constraints. It is obviously impossible to generalize the results to the whole country. However, the objective of this study is not to make statistical inferences but to try to understand in depth how this maternal health policy was implemented in six health centres. In this sense, the heuristic value of the contrasted cases (health centres) was the priority. The findings of this study are similar to findings from other districts and other countries [32]. Therefore, these similarities lead to a “*theoretical generalization*” about the phenomenon under study [12].

## Conclusions

Removing user fees for health care is an ambitious political policy. The impact of this type of policy on service utilization and out-of-pocket expenditures is becoming well known. However, implementation evaluation is still scarce. This article contributes to our understanding of the implementation process of this policy. It highlights the challenges faced by implementers and provides an overview of how this maternal health policy was implemented and why it was done in this way. The “*implementation gap"* analysis has shown that equity and quality of care were neglected and should be taken into consideration. Moreover, it raises interactional factors that have an influence on the process of implementation. This study has shown the importance of considering power relations between actors representing health system and communities. While power relations have been recognized as a key factor in health policy analysis, explicit analysis is still scarce. In the context of improving financial access to maternal health care, more studies should be encouraged to examine power relations within the implementation process.

## Competing interests

The authors declare no competing interests.

## Authors’ contributions

Both authors participated in the study design. LB collected and analyzed the data and wrote the first draft under the supervision of VR. Both authors read and approved the final draft.

## Pre-publication history

The pre-publication history for this paper can be accessed here:

http://www.biomedcentral.com/1471-2393/12/143/prepub
